# A Robotic Teleoperation System Enhanced by Augmented Reality for Natural Human–Robot Interaction

**DOI:** 10.34133/cbsystems.0098

**Published:** 2024-04-08

**Authors:** Xingchao Wang, Shuqi Guo, Zijian Xu, Zheyuan Zhang, Zhenglong Sun, Yangsheng Xu

**Affiliations:** ^1^ Shenzhen Institute of Artificial Intelligence and Robotics for Society (AIRS), The Chinese University of Hong Kong, Shenzhen, Guangdong, China.; ^2^ School of Science and Engineering, The Chinese University of Hong Kong, Shenzhen, Guangdong, China.; ^3^ Dyson School of Design Engineering, Imperial College London, London, UK.

## Abstract

Telekinesis, as commonly portrayed in science fiction literature and cinema, is a super power wherein users control and manipulate objects absent in physical interaction. In real world, enhancing human–robot interaction needs the synthesis of human intuitive processes with robotic arm. This paper introduces a robotic teleoperation system achieving the essence of telekinetic operations, combining the profound capabilities of augmented reality (AR) with the robotic arm operations. Utilizing AR, the proposed methodology offers operators with a visual feedback, facilitating a level of control surpassing the capacities of natural interfaces. By using AR-driven visual recognition, this system achieves operations in a virtual environment, subsequently actualized in the real world through the robotic arm. Through multiple experiments, we found that the system has a small margin of error in telekinesis operations, meeting the needs of remote operation. Furthermore, our system can operate on objects in the real world. These experiments underscore the capability of the remote control system to assist humans in accomplishing a wider range of tasks through the integration of AR and robotic arms, providing a natural human–robot interaction approach.

## Introduction

Telekinesis, often mentioned in science fiction novels and movies, is a supernatural ability where individuals can manipulate objects solely through their thoughts, without the need for physical contact. The transition of this sci-fi notion into practical applications has become a hot topic in the field of human–robot interaction. Achieving telekinesis has the potential to enhance human–robot interaction capabilities, enabling individuals to remotely perform challenging tasks while the robot executes them directly.

Teleoperation [1] has traditionally served as an effective means of controlling robots, effectively bridging the complexity of robot-driven tasks with human intuition. However, previous remote robot operations were confined to using physical controls, requiring unnatural input devices such as keyboards or joysticks [2]. In these traditional robot teleoperation systems, while their interfaces were feature-rich, users often needed substantial time to learn their operation due to the lack of intuitive visual feedback. Consequently, a more natural human–robot interaction necessitates more direct and natural visual feedback. Nonetheless, with the rapid advancement of technology, augmented reality (AR) [3] has emerged as a more immersive and intuitive medium for interaction. AR-based teleoperation offers a more intuitive and natural means of remote control.

In recent years, AR has become a prominent technology, enriching human perception and interaction. The potential of teleoperation combining AR and robotics is boundless. By introducing an additional layer of visual feedback through AR technology, it theoretically addresses many inherent challenges in traditional remote operations. Furthermore, the fusion of AR and robotics offers a more natural mode of interaction, potentially minimizing the cognitive load on operators while maximizing precision in operations. However, seamlessly integrating AR and robotics for telekinesis presents challenges [4]. Ensuring seamless communication between these 2 domains, guaranteeing real-time responsiveness, and translating virtual interactions into precise robotic actions are critical hurdles to overcome.

**Fig. 1.**
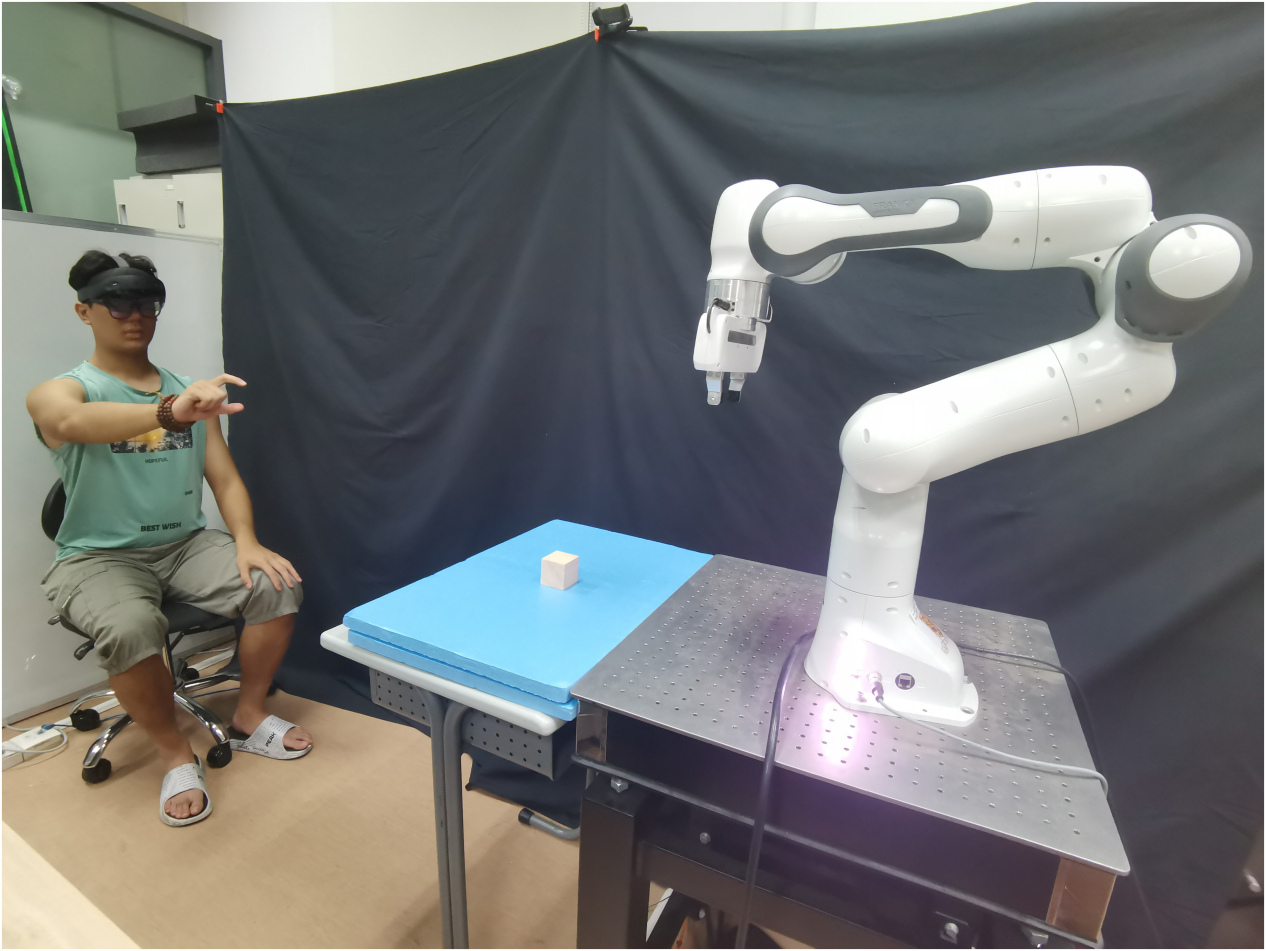
A robotic teleoperation system enhanced by AR for natural human–robot interaction.

Therefore, this paper proposes an innovative teleoperation system-merging the immersive capabilities of AR with the mechanical precision of robotic arms, as depicted in Fig. 1. The introduction of AR brings a fresh perspective to this system. AR overlays computer-generated images onto the user’s view of the real world, bridging the perceptual gap between humans and robots. By presenting digital information in the real world, AR technology serves not only as a visual aid but also as an advanced means of human–robot collaboration. This research focuses on the synergy between AR’s immersive experience and the mechanical precision of robotic arm teleoperation. Users can manipulate real-world objects in a virtual environment and send commands to the robot arm, which can execute these actions in the actual environment. The ultimate goal is to enable individuals to interact with objects remotely without physical contact. The combination of AR’s immersive experience and the mechanical flexibility of robotic arms represents a pioneering approach, allowing for unprecedented levels of precision and control.

Our proposed system provides a more direct and intuitive framework through the visual feedback offered by AR. Users can operate objects remotely based on their real-world interaction habits, aligning with their intuitive understanding. This approach reduces the user’s learning curve, potentially enhancing the accuracy and efficiency of operations. In addition, leveraging AR’s visual recognition capabilities enables a smoother transition between operations performed in the virtual domain and physical implementations executed by the robotic arm. This seamless connection not only enhances operational efficiency but also represents a paradigm shift in how we perceive and interact with robotic systems. By allowing users to interact with remote environments through familiar real-world gestures and actions, our system transforms the teleoperation experience into a more user-friendly and efficient process. The fusion of AR’s visual feedback and robot control opens up exciting possibilities for a wide range of applications, from industrial automation to medical procedures, enhanced human–robot cooperation, and expanding the scope of what we can achieve in remote control fields.

The remaining structure of this paper is as follows. First, we delve into the relevant research on AR-based remote operations. Next, we introduce the proposed robotic remote operation system, encompassing the AR headset, the robotic arm, and the communication protocols. We then detail the methods and interaction framework used for remote operations. Subsequently, we validate the accuracy of the system’s operations and the effectiveness of the method through a series of experiments. Multiple real-world experiments are conducted to demonstrate the system’s capability across various tasks. Finally, we discuss the research findings and conclude this paper.

## Related Work

### Robotic teleoperation

Robotic teleoperation is a method of remotely controlling robots, and numerous researchers have explored various approaches to this field of study. A method for semiautonomously teleoperating a robot arm is proposed, incorporating joint-space control and autonomous end-effector position control for the entire arm [5]. A technique to enhance remote users’ teleoperation capabilities is proposed by providing them with a continuous and effective viewpoint using a robot arm equipped with a camera [6]. An innovative haptic shared control paradigm is proposed that adapts the level of robotic guidance based on predictions of human motion intentions [7]. Comprehensive review of Wonsick and Padir [8] classifies research on virtual reality interfaces in robot operation into visualization, control, interaction, usability, and infrastructure, highlighting the role of virtual reality in intuitive human–machine interfaces. Feng et al. [9] developed a teleoperated robot system with haptic feedback for microinjection, enhancing task success through an interface that simulates the real feeling of injection, demonstrating the effectiveness of tactile feedback in precise operations. Caiza and colleagues [10] explored teleoperation systems for oil field maintenance, adopting distributed control schemes and lightweight communication protocols for efficient and safer operations in hazardous environments. Yang et al. [11] proposed a teleoperation mechanism using surface electromyography and haptic feedback, mimicking human muscle activation, significantly enhancing robot manipulability and task execution, and paving the way for more intuitive human–machine interaction. Kaplish and Yamane [12] addressed challenges in teleoperated physical human–robot interaction, developing novel algorithms for motion retargeting and control. This system effectively tracks force commands while maintaining the operator’s motion style, aiding in more accurate and intimate physical interactions in teleoperation. Qi et al. [13] proposed a multisensor-guided hand gesture recognition system for surgical robots, using an Long Short-Term Memory Recurrent Neural Network (LSTM-RNN) model that offers higher gesture classification accuracy and efficiency, highlighting potential in surgical applications. The study of Hassan and colleagues [14] on controlling robotic arm movement using surface electromyography (sEMG) signals with a Myo armband revealed the high accuracy of the support vector machine classifier in real-time control, indicating the potential of sEMG signals for intuitive control. The model-based teleoperation architecture of Kazanzides et al. [15] for remote space intervention emphasizes the importance of enhanced visualization and precise control in overcoming communication delays. Darvish and team’s [1] survey on teleoperation of humanoid robots underscores their versatility and the necessity of human involvement solutions in complex and dynamic environments, emphasizing the integration of human cognitive skills with robotic capabilities in hazardous settings. Battisti and Muradore’s [16] implementation of the Optimal Reciprocal Collision Avoidance (ORCA) method for unmanned aerial vehicle–unmanned ground vehicle interaction and force feedback in remote operation showed improved collision avoidance and control in dynamic environments, further enriching the field of robotic teleoperation. However, most visual feedback in robotic teleoperation is presented to users through screens. With technological advancements, virtual and AR have significantly enhanced visual feedback in remote control applications.

### AR-based operator system

AR is a novel interactive method created by display and visual processing technologies, allowing the overlay of virtual world information onto the real world, transforming virtual control into reality. In the realm of human–machine collaboration, visual methods applied by AR mainly include head-mounted displays, spatial AR projectors, fixed screens, and handheld displays [17]. The current research trend in AR primarily focuses on conveying the intent of controlling robotic arm movements through mixed reality headsets. A visual solution for mixed reality headsets has been proposed, displaying robots and their surroundings in the wearer’s real-world context [18]. Another study introduced a workspace recognition model based on depth sensors and an interactive AR user interface to enhance human labor productivity safely [19]. An AR tool has been developed to assist operators in working within mixed industrial environments [20]. By utilizing machine learning to identify tasks being performed by operators and enabling robots to collaborate on shared tasks, it provides customized operator support that adapts to their needs and preferences [21]. In addition, a robotic user interface system was created, incorporating AR, visual feedback, and tactile control to facilitate intuitive and efficient collaboration between users and robotic arms [22]. Regal et al. [23] studied the use of AR in hot cells for remote operation of a 2-arm manipulator, demonstrating the potential of AR in improving safety and operating efficiency. Zhang et al. [24] explored combining the FreeForm haptic system and AR technology to improve tactile simulation in space remote operations and proposed the FreeForm robot arm as a remote control execution device concept. Poignant’ team [25] explored new methods of combining virtual robotic arms with physical mechanical manipulators, providing a new perspective on wearable assistive robotics technology. McMullen’s team [26] designed an AR-based control system for the 3-dimensional end of the robotic arm, the direction of the gripper, and the gripper diameter. There are many studies on AR operating robotic arm systems in the field of surgery. D’Amato et al. [27] discussed the application of AR headsets in surgery, emphasizing its importance in improving surgical accuracy and efficiency. Lee et al. [28] proposed a skin-conforming, stretchable, and breathable marking patch for AR surgical navigation systems, which improved positioning accuracy during surgery. In the field of smart device interaction, Becker’s team [29] studied different interaction methods for controlling smart devices in AR environments, including gestures and the use of physical objects. It provides a new perspective for the interaction between AR and robotic arms by discussing the method of wearable AR devices to control smart devices [30]. Many works focus on using AR to help users visualize when operating the mechanical arm. Zhou et al. [31] produced a set of robotic arm visualization applications based on the interaction of virtual buttons in AR. This paper primarily focuses on remotely controlling a robotic arm without physical contact, using AR glasses for feedback control. The system leverages the advantages of AR’s intuitive feedback and provides a natural mode of operation for achieving teleoperation.

## System Overview

**Fig. 2.**
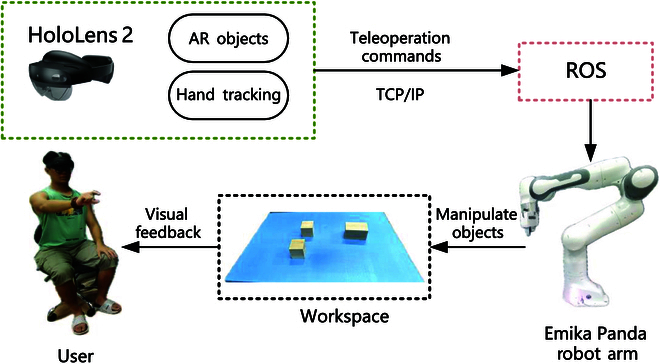
System overview of the robotic teleoperation system enhanced by AR.

The system consists of an AR headset, control system, and an Emika Panda robot arm, as shown in Fig. 2. The AR headset captures the environment in real time, processes it, and overlays virtual controls and data. The robot arm manipulates the objects based on the teleoperation commands transmitted by communication method.

The AR headset serves to capture distant objects and environments for creating a virtual environment and generating virtual objects based on recognized items. Hand tracking is used for monitoring hand movements, recognizing gestures, and controlling the manipulation of virtual objects. In addition to overlaying digital information onto the real world, the AR device we use is lightweight and comfortable, ensuring that users can use it for extended periods without fatigue. The robotic arm serves as our primary means of manipulating real-world objects by sending commands.

Notably, the true innovation of our system lies in the integration of the physical and virtual realms, combining AR and robotic arm functionalities. Our algorithmic approach combines the robotic arm and AR device. After modeling data from the real world, it is overlaid onto the operator’s view. Our AR device is equipped with cameras and motion sensors to capture the operator’s movements and gestures. When the operator points to a location or makes specific gestures, our algorithm interprets these actions and sends corresponding commands to the robotic arm. This smooth bidirectional interaction assists the operator in guiding the robotic arm’s movements, all in real-time.

### AR headset

Microsoft’s HoloLens 2 [32], a cutting-edge AR headset, offers a range of technical specifications that are particularly pertinent to its integration into a robotic teleoperation system. HoloLens 2 uses advanced waveguide optics to deliver high-resolution holograms seamlessly integrated into the user’s field of view. This technology ensures that virtual objects appear naturally within the physical environment, enhancing the realism of human–robot interactions. It offers an expanded field of view compared to its predecessor, enabling users to perceive a broader context. This wide field of view enhances situational awareness, a crucial factor for effective teleoperation of robots in complex environments. Hand tracking capabilities enable users to interact with holographic content using natural hand gestures. These intuitive controls can be harnessed to issue commands and manipulate objects in the robot’s environment, fostering a more intuitive control paradigm. HoloLens 2 facilitates the capture of mixed reality footage, combining holographic content with real-world video. This capability is valuable for recording and analyzing teleoperation sessions, aiding in the evaluation and improvement of human–robot interactions. The device creates a 3-dimensional representation of the environment and objects. This information helps to assist the user in constructing a virtual replica of the real world and enables commands to be issued to control real objects by remotely manipulating the virtual objects.

### Robot arm

Emika Panda [33] is a versatile and advanced robotic arm designed for a wide range of applications in industrial and research settings. Emika Panda is equipped with 7 degrees of freedom, allowing for complex and precise manipulations. This extensive range of motion enables the robot to perform intricate tasks with flexibility and accuracy. The robot arm is suitable for handling a variety of objects and tools in industrial processes and research experiments. Its workspace is optimized for efficient task execution in confined spaces while maintaining a wide operational range. The robot arm offers high repeatability and accuracy. This level of precision is essential for tasks requiring tight tolerances and consistent results. The robot arm supports a range of communication protocols and interfaces, enabling seamless integration into existing automation systems and research setups. It can also be controlled remotely for enhanced versatility. The Emika Panda is an adaptable robotic arm that is able to manipulate objects that need to be operated remotely and with a high degree of precision by means of the gripping jaws of the end-effector.

### Teleoperation communication

This section presents an overview of the technical specifications related to the Transmission Control Protocol/Internet Protocol (TCP/IP) communication [34] established between Microsoft’s HoloLens 2 AR headset and a robot arm utilizing the robot operating system (ROS). HoloLens 2 is equipped with advanced Wi-Fi capabilities, allowing it to establish a reliable wireless connection to the local network. This wireless link is essential for real-time communication with the ROS-based robot arm. The Emika Panda robot arm incorporates ROS middleware, which enables the creation of ROS nodes for communication. ROS nodes are responsible for handling data transfer and command execution, facilitating seamless integration with the HoloLens 2. TCP/IP is utilized as the communication protocol between HoloLens 2 and the robot arm. TCP ensures reliable and ordered data transmission, which is crucial for controlling the robot arm accurately and receiving real-time feedback.

The communication protocol defines standardized data formats for transmitting various types of information. This includes the object’s position and orientation. To ensure that commands issued by the user via HoloLens 2 are synchronized with the robot’s actions, synchronization mechanisms are used. Timestamping and acknowledgment protocols are implemented to maintain precise control. The technical specifications associated with TCP/IP communication between HoloLens 2 and a robot arm running ROS are integral to establishing a reliable, low-latency, and secure link for natural human–robot interaction.

## Methods

In this section, we delve into the core methods and algorithms pivotal to the establishment of a comprehensive virtual environment, enabling users to recognize gestures and interact with virtual objects seamlessly. In addition, we elucidate an effective coordinate transformation technique, facilitating synchronized motion between the virtual environment and the physical robot arm. Finally, we provide an in-depth description of the implementation of these methods and technologies, culminating in the construction of a holistic interaction framework. This framework allows users to engage with both the virtual environment and the real robotic arm through a series of carefully crafted steps.

### Virtual AR objects

**Fig. 3.**
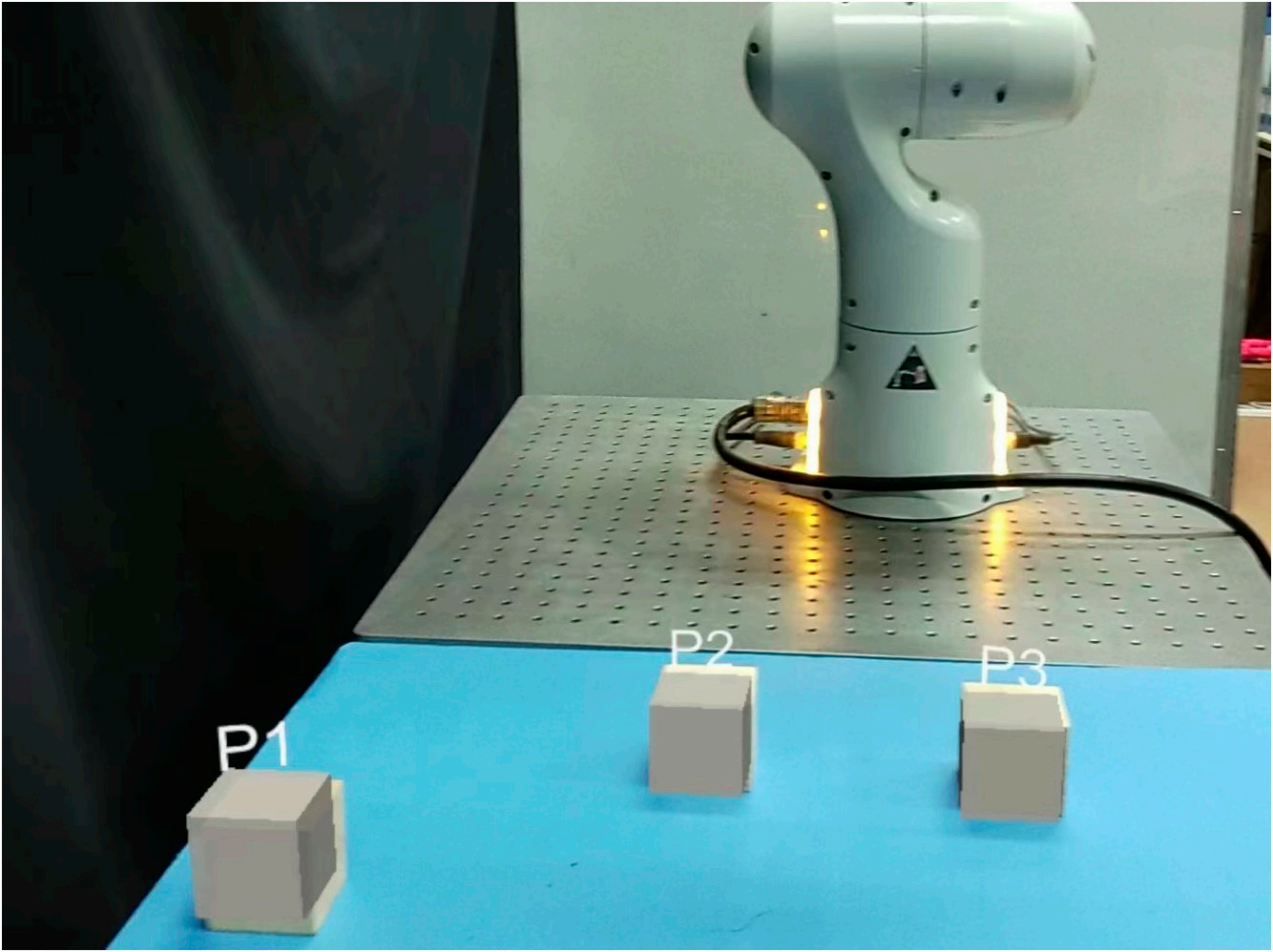
In the perspective of HoloLens 2, a virtual coordinate system is established on the basis of the real world, and real objects are identified to create virtual objects. In the diagram, P1, P2, and P3 represent different cubes, which users can interact with in the virtual environment.

HoloLens 2 utilizes its spatial mapping capabilities and sensors to understand and map the real-world environment. This involves scanning the surroundings, detecting surfaces, and creating a spatial mesh that represents the physical space. Once the spatial mapping is complete, the system aligns the virtual coordinate system with the real-world coordinates. An object recognition method is integrated into the system, capable of real-time recognition of objects within the workspace based on their shapes. When a real object is recognized, the device associates it with a specific position in the virtual coordinate system. On the basis of the recognized real-world objects, the system generates virtual objects that correspond to them. These virtual objects are superimposed onto the physical environment, aligning with their real-world counterparts. Users can see and interact with these virtual objects as if they were part of the real world, as shown in Fig. 3.

### Gesture recognition

**Fig. 4.**
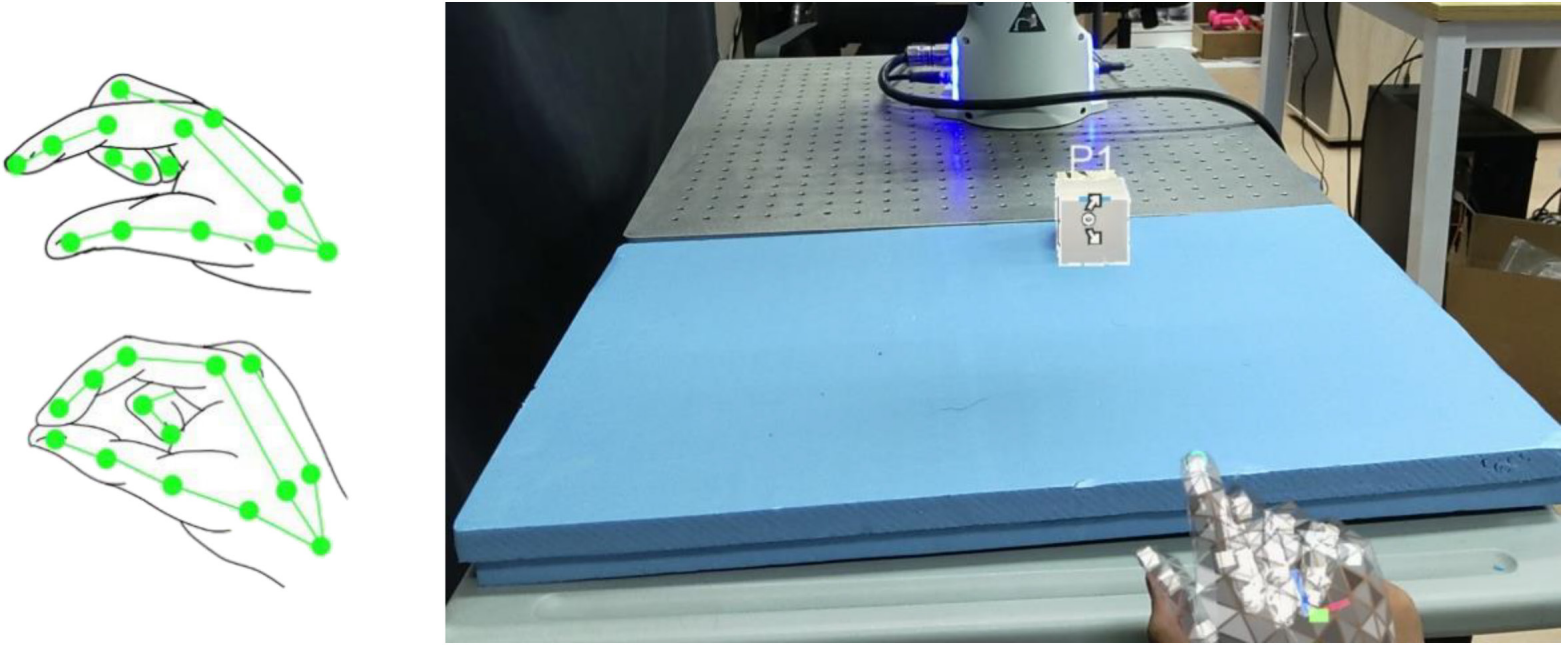
Left: The gesture recognized by algorithm [35]. Right: In the perspective of HoloLens 2, the system recognizes gestures and specifying the manipulation of a virtual cube.

To facilitate the manipulation of virtual blocks, this system requires the capability to recognize gestures. We have used a one-dimensional convolutional neural network structure, as described in [35], to train with the multiview multitask paradigm. This algorithm leverages multiple time-local views of gestures and actions to generate a posture description and defines multiple task-specific gestures, as shown in Fig. 4. The system enables users to interact with virtual objects naturally. Users can use hand gestures to manipulate and control these virtual objects. For example, users can reach out and grab a virtual cube and move it within the virtual coordinate system. Users can select virtual objects by pinching with their fingers and manipulate them by dragging to the desired location. The system records the actions performed on the virtual objects, including their initial and final states, to transmit to ROS.

### Coordinate conversion

To facilitate the interaction between HoloLens 2 and the robotic arm, enabling the sharing of virtual and real spatial data, we introduce a method for aligning the virtual spatial coordinate system with the real spatial coordinate system. This alignment is essential to establish a seamless connection between these coordinate systems.

Given that HoloLens 2 primarily focuses on target tracking within the AR system, its coordinate system is initially set on the basis of the device’s position at power on. To achieve accurate tracking, we use a marker-based approach within the virtual world. Once the virtual world calibration is performed after powering on the system, recalibration of the virtual world coordinate system is rarely required since it remains fixed.

**Fig. 5.**
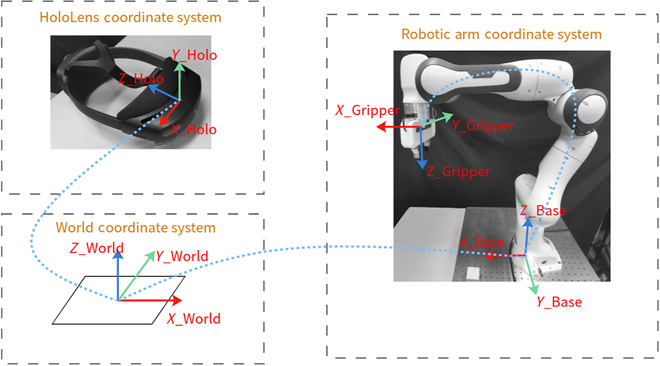
HoloLens 2 virtual space, world coordinate system, mechanical arm coordinate system, and coordinate system transformations.

To bridge the virtual spatial coordinate system with the real spatial coordinate system of the robotic arm, we devise a coordinate transfer method. As shown in Fig. 5, this method leverages the position and orientation of the “virtual object” presented by the AR as an intermediary medium. Since the robotic arm remains stationary within this system, we establish the base coordinate system of the robot base in advance, along with the relationship between the origin and the base. The alignment between the origin of the virtual spatial coordinate system and the real spatial coordinate system of the robot arm is determined on the basis of this intermediary. In addition, the end-effector’s coordinate system is derived using the forward kinematics of the robotic arm. The specific calculation method is as follows. ${}_{\text{target}}^{\text{base}}T$: Transformation matrix from the target coordinate system to the base coordinate system. *R* and *T* are initial values that need to be set on the basis of the startup position of HoloLens 2 each time it begins.

$$\left[\begin{array}{c} X_{\text{Base}}\\ Y_{\text{Base}}\\ Z_{\text{Base}}\\ 1 \end{array}\right]=\left[\begin{array}{cc} R & T\\ 0 & 1 \end{array}\right]\left[\begin{array}{c} X_{\text{World}}\\ Y_{\text{World}}\\ Z_{\text{World}}\\ 1 \end{array}\right]$$ (1)

$${}_{\text{target}}^{\text{base}}T{=}_{\text{target}}^{\mathrm{AR}}T\cdot_{\mathrm{AR}}^{\text{world}}T\cdot_{\text{world}}^{\text{base}}T$$ (2)

Hence, utilizing the coordinate system construction method described above, we can establish communication between the AR target information from HoloLens 2 and the robotic arm, facilitating communication following coordinate transformations in practical applications.

### Interaction framework

**Fig. 6.**
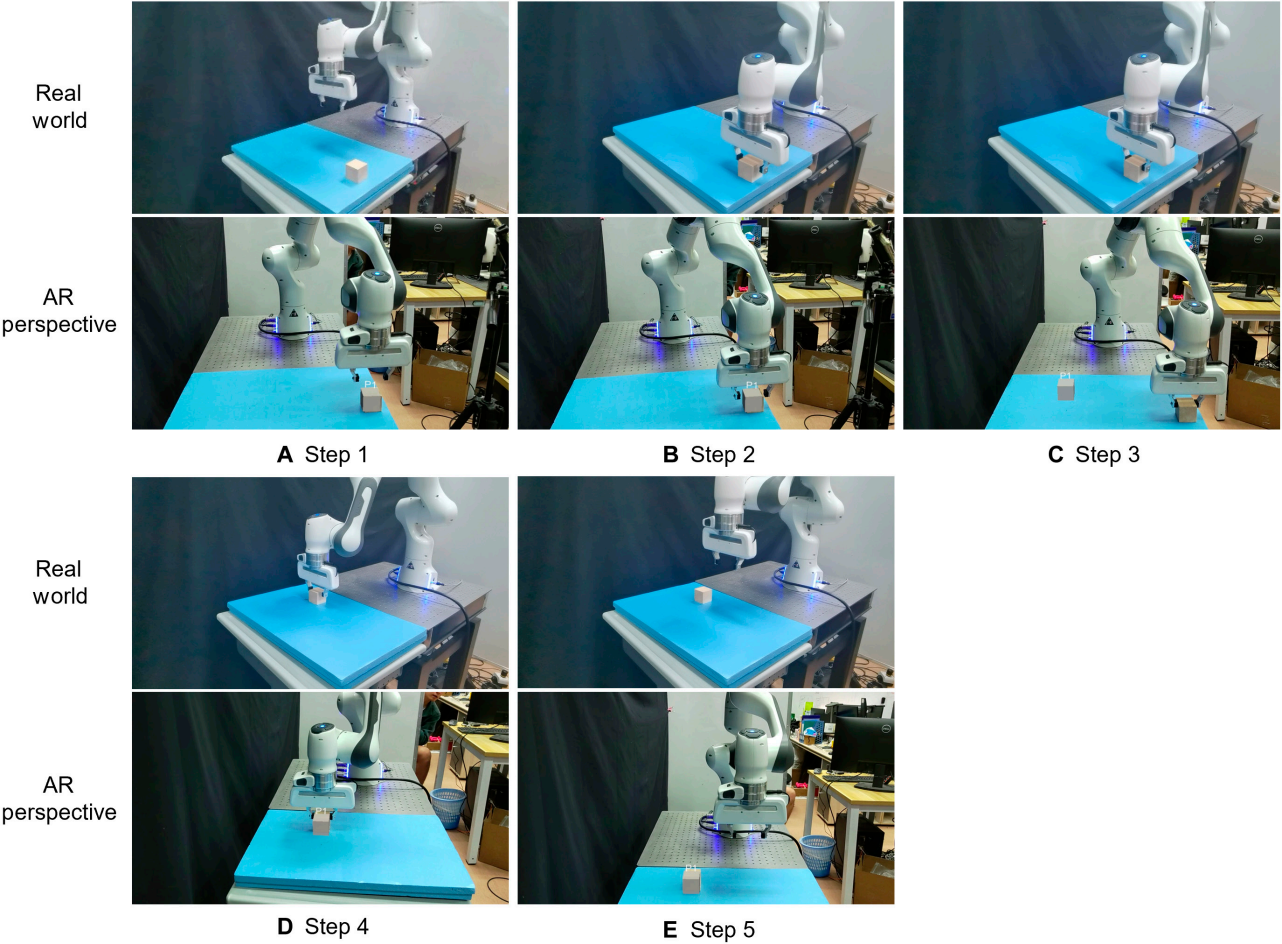
Interaction framework: The interaction framework can be simplified into (A to E) 5 steps to achieve the goal of controlling a robotic arm to manipulate real objects remotely through the manipulation of virtual objects.

A user-friendly interaction framework is introduced in this section, amalgamating the aforementioned methods and technologies. This framework defines clear interaction steps encompassing gesture recognition, virtual object selection and manipulation, coordinate transformation, and robotic arm motion execution. By integrating these elements, we construct a comprehensive virtual–human interaction system, tightly integrating the virtual environment, gesture recognition, and real robotic arm control. As shown in Fig. 6, the interaction framework can be simplified into 5 steps to achieve the goal of controlling a robotic arm to manipulate real objects remotely through the manipulation of virtual objects. "The process of remotely moving a block from one location to another is illustrated in Fig. 6.

1. Identifying and confirming the object to be manipulated through gesture recognition, followed by transmitting commands to the ROS control system through communication.
2. The robotic arm receives the command to grasp the cube.
3. The user manipulate the virtual object through gestures, moving it to the desired location, and confirming the action, which then transmits the target command to the robotic arm system.
4. The robotic arm receives the command and subsequently plans a trajectory to move to the target position.
5. The user continues the operation, and the robot can standby for the next command.

## Results

In this section, we conducted several experiments, encompassing both quantitative and qualitative aspects. Our primary objective was to thoroughly evaluate the system’s precision and usability, gaining a comprehensive understanding of its performance in different scenarios and under different conditions. Through this multifaceted approach, we aimed to provide a holistic assessment of the system’s capabilities and its potential practical applications.

In our experiment, the hardware setup comprised a HoloLens 2 AR headset, a Franka Emika Panda robotic arm and a desktop computer running the Linux operating system. This configuration was essential for facilitating communication and relaying control signals to the robotic arm. The control of the robotic arm was orchestrated through the ROS platform on Linux. This setup was crucial for processing control commands sent from the HoloLens 2 via a local area network. The ROS platform acted as an intermediary, receiving these commands and then transmitting them to the robotic arm. This integration enables precise and responsive control of the robotic arm based on the user’s inputs through the HoloLens 2.

### Error estimation

To comprehensively assess the system’s stability and accuracy, we designed and conducted experiments consisting of 5 sessions, with each session involving 10 repeated trials. These experiments were carried out to ensure that the system’s performance consistently met the high standards we set for it.

**Fig. 7.**
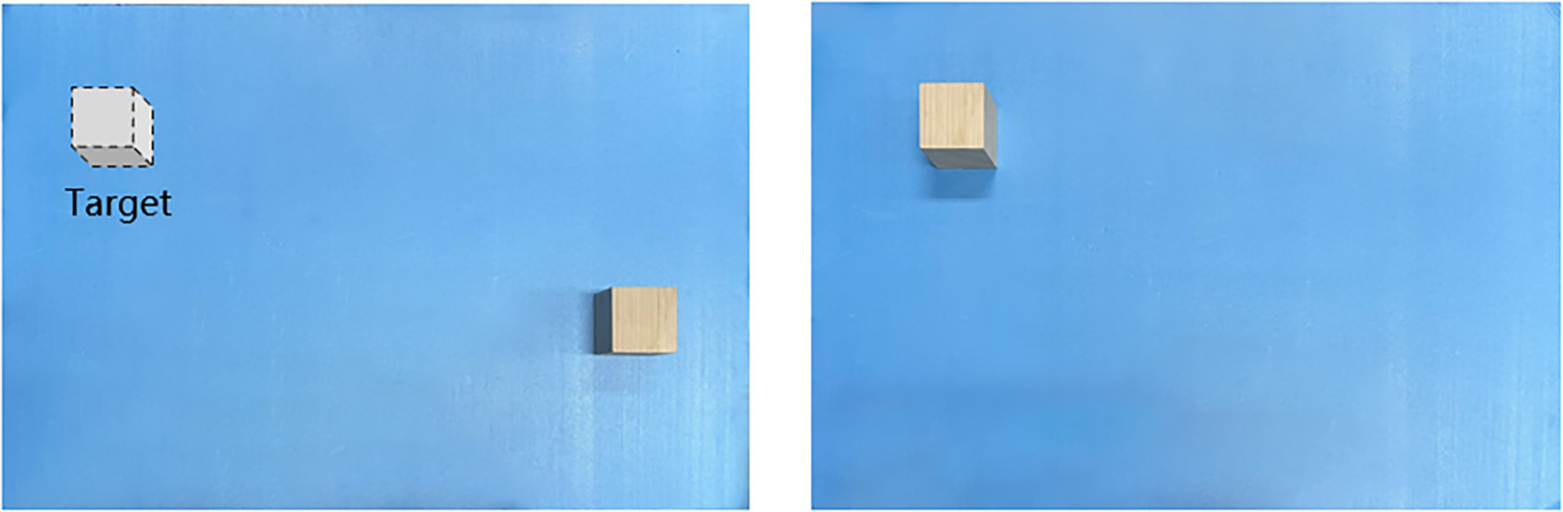
The setup of error estimation experiments.

These experiments revolved around the repetitive movement of virtual objects, such as blocks, with the objective of precisely positioning them at target locations. As depicted in Fig. 7, this represents the experimental setup for one session, where wooden blocks were moved to their target positions. After each movement controlled through AR, we measured the error between the actual and target positions of the blocks. Since this movement occurred in a plane, only errors in the *x* and *y* directions were measured, as there was no movement in the *z* direction. This measurement process allowed us to quantitatively assess the accuracy of our system’s operations.

To ensure the reliability of our results, we conducted a substantial number of trials for each experiment, altering the initial positions of the blocks and their target positions in each session. In total, we conducted 5 distinct sessions, with 10 repeated trials in each session, from which we derived errors and calculated their averages. This diverse range of experiments provided a robust dataset, enabling us to compute relative errors. By taking the average of these errors, we obtained a reliable measure of the overall accuracy of the system’s performance. As indicated in Table, the errors were consistently controlled within 0.35 cm, meeting the requirements of our remote control system.

**Table.** Errors of the system

		*x*	*y*
Error (cm)	Session 1	0.2370	0.2864
	Session 2	0.3358	0.2395
	Session 3	0.2123	0.2148
	Session 4	0.2677	0.1414
	Session 5	0.3012	0.2333

### Applications in the real world

**Fig. 8.**
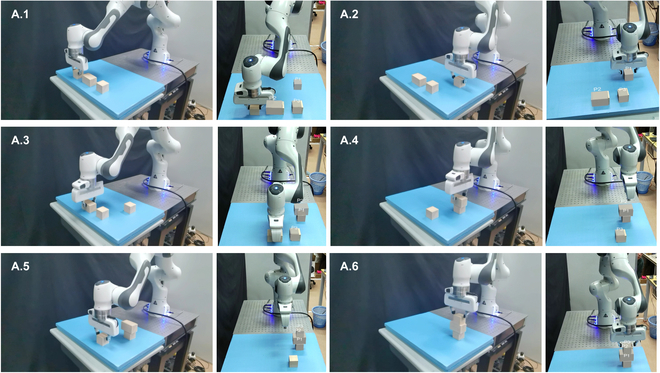
Stacking blocks game experiment. Left: Real world manipulation of robot arm. Right: Perspective of AR headset.

**Fig. 9.**
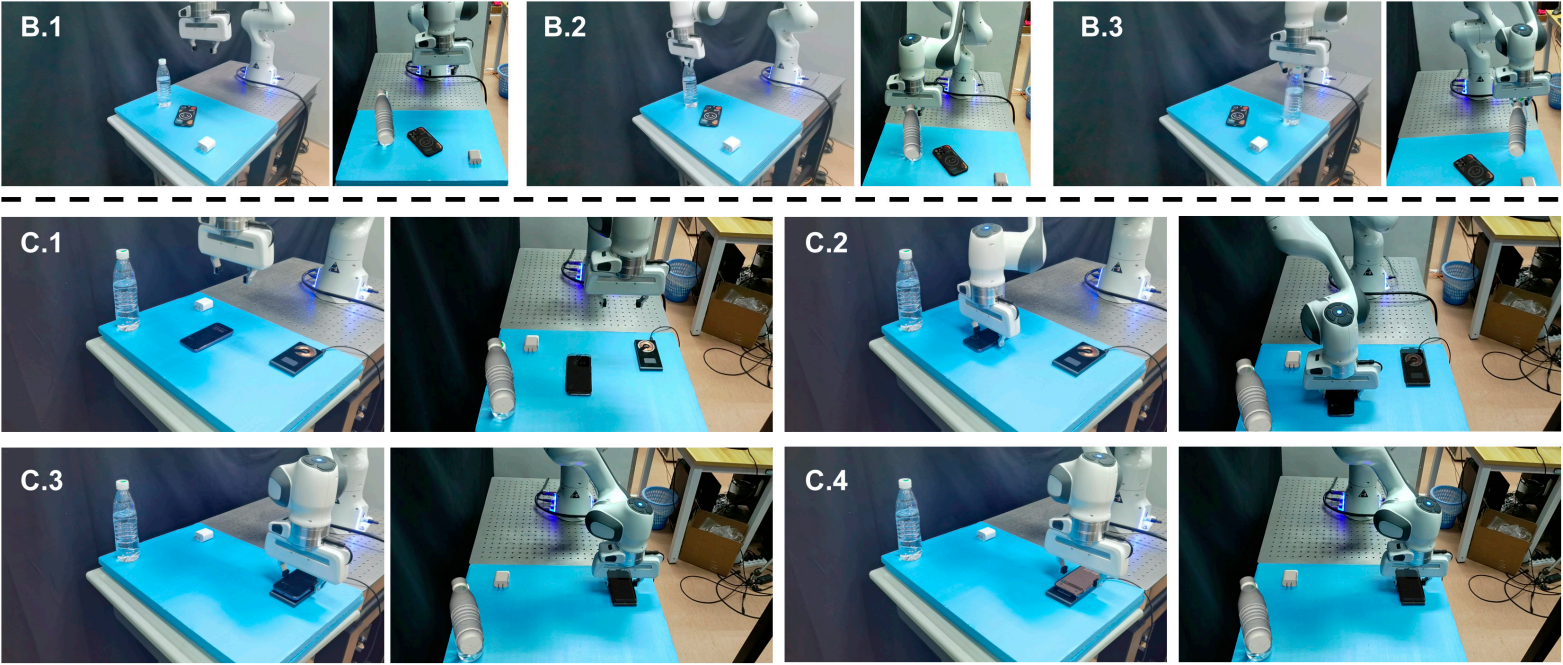
Applications in the real world. B.1 to B.3 show the grasping of a water bottle and placing it in a designated location. C.1 to C.4 show assisting the user in moving the phone to the wireless charger and successfully charging it. Left: Real world manipulation of robot arm. Right: Perspective of AR headset.

In this experimental section, we engaged our test participants in a series of telekinetic tasks within real-world scenarios, utilizing the capabilities of our system. The first task presented to our participants involved the manipulation of 3 blocks in a block-stacking game. This task is of significant importance, especially in the context of rehabilitation for patients dealing with injuries or motor skill development. In the second task, our participants were tasked with the remote operation of a mobile phone that was running out of battery, requiring them to perform wireless charging. This scenario reflects a common real-world situation where remote control can be advantageous. In the third task, participants were asked to move a bottle, showing the versatility of the system in practical, everyday activities. All experiments were conducted with the participants positioned at a distance of 1 m from the workspace, facing the robotic arm and benefiting from real-time visibility of the arm’s operations. The successful completion of all 3 experiments underscores the effectiveness and adaptability of our system. Figures 8 and 9 provide snapshots capturing key moments during each experiment, illustrating the seamless execution of these tasks through our telekinesis technology.

While all users were able to successfully complete the experiments, testers occasionally made multiple adjustments to ensure precision in their operations. The intuitiveness of AR brings excellent correction capabilities, allowing testers to intuitively observe the robotic arm’s movements and the success of their tasks. This human-centric approach provides a more natural human–robot interaction capability. For instance, in the case of the wireless charging task, the wireless charging module required precise placement in a specific area to complete the experiment. Initially, users could place the phone on the charging pad but could not initiate charging. However, after several adjustments, testers were able to accurately position the phone on the charging pad and commence charging. In addition, users might need to establish the initial and target states and carefully manipulate the robot arm to ensure effective control between the arm and the object. Although this system also requires users to adapt, the adaptation time and learning curve are significantly reduced because of its intuitive nature.

**Fig. 10.**
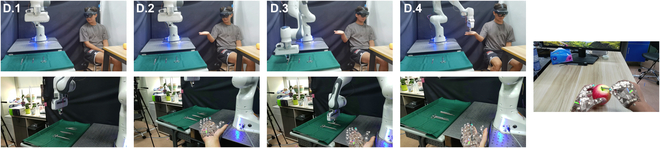
Surgical instrument handover in a medical setting. Top: Real-world manipulation of robot arm. Bottom: Perspective of AR headset.

We discovered that besides facilitating everyday scenarios, our system also excels in specific settings. For instance, in medical environments such as operating rooms, doctors and nurses often collaborate to perform surgeries. Instrument handover is a critical task for nurses. Therefore, as illustrated in Fig. 10, we designed an experiment simulating the interaction of surgical instruments between doctors and robotic nurses. Initially, the doctor selects the required surgical instruments (such as forceps, scalpels, etc.) using AR technology. Then, by simply extending their hand, the robotic arm places the instrument in the doctor’s palm, ready for use in surgery. This system can partially replace the role of nurses in the operating room, thereby alleviating some surgical pressures. To explore this application, we set up a collaborative robot as the robot nurse, and, as shown in Fig. 10, it depicts the workflow:

1. The user uses AR to select needed instruments such as tweezers and scalpels.
2. As the user reaches out, the headset identifies human gesture and sent corresponding commands to the robot.
3. The robot executes the command by picking up the desired surgical instrument.
4. The robot hands over the instrument to the human user.
5. The user continues the operation, and by identifying the surgical task flow, the robot can standby for the next command.

## Discussion

On the basis of our experimental findings, the robotic teleoperation system equipped with AR can successfully complete handover tasks, demonstrating a range of potential applications. This integration of AR and robotic arms proves beneficial in several fields, each leveraging its unique capabilities. In the medical field, the system revolutionizes surgical procedures. Surgeons can intuitively view and interact with the operational area using the AR interface. This advancement not only simplifies the learning and operation of surgical control systems but also increases the accuracy and safety of procedures through enhanced visual feedback. In addition, the system’s ability to undertake roles typically performed by nursing staff can greatly alleviate workloads during extended surgeries. Similarly, in the industrial field, the system’s utility is highlighted in tasks that require collaboration with robotic arms, especially in handling hazardous materials or performing high-precision operations. The system’s intuitive design, combined with its capability to interpret human intentions, renders it an invaluable tool in various industrial processes, thereby improving safety and efficiency. Likewise, in the space exploration, its intent recognition capabilities can support collaborative efforts with astronauts, proving particularly beneficial in the exploration and maintenance of space stations. This assistance in complex tasks could potentially reduce the risks associated with extraterrestrial missions. Overall, these diverse applications underscore the system’s potential to transform operations across multiple domains, from enhancing medical procedures to improving industrial efficiency and space exploration.

Our system currently faces several limitations, which we are actively working to address to en4hance its performance. A primary limitation is the restricted field of view in the AR headset. We are addressing this by exploring the use of more advanced optical components and image rendering technologies. Our goal is to expand the users’ environmental perception, thereby enhancing the precision and efficiency of operations. Another significant limitation lies in the current inadequacy of the accuracy and speed of object recognition. This aspect is crucial for intuitive and natural remote control. To improve this, we plan to enhance the system’s recognition capabilities, especially in complex environments, with the aim of boosting both responsiveness and reliability. Furthermore, the limited workspace of the robotic arm is a notable constraint on the system’s applicability. To address this, we intend to install the robotic arm on a mobile platform, which will enable precise operations over a larger area. Last, the stability and response speed of the system are often compromised by network delays or interference. To mitigate this issue, we are investigating the adoption of more advanced network technologies, such as 5G or dedicated network connections. We anticipate that these advancements will reduce latency and improve the reliability of data transmission, thereby enhancing the overall performance of the system.

## Conclusion

This paper introduces an AR-based remote control system designed for natural human–robot interaction. The system comprises an AR headset and a robotic arm, enabling a wide range of operations within a large workspace. Consequently, users can leverage AR for creating virtual environments and manipulating virtual objects while also interacting with physical objects in the virtual environment. The robust capabilities of the robotic arm allow for precise and intricate manipulation of objects, minimizing errors. Furthermore, because of AR’s ability to overlay virtual environments onto the real world, the human–robot interaction is more intuitive, aligning with human intuition. We conducted various test experiments to verify the system’s low error rates in operations, demonstrating its versatility across real-world tasks.

However, there are still some challenges to address for future developments to make the system even more practical. First, because of limitations in the field of view of HoloLens 2, improvements in object and coordinate recognition accuracy are needed, particularly for smaller objects. Consideration can be given to integrating external cameras to enhance recognition accuracy. Second, although the current robotic arm has the ability to perform basic operations such as grasping, it falls short of replicating the fine motor skills of a human hand. In the future, replacing the gripper with a dexterous hand as the end effector could enable more precise actions. Last, the system currently supports single-hand recognition and operation. Enabling dual-hand operation could significantly enhance the speed and complexity of object manipulation, further advancing the telekinesis system.

## Data Availability

As this paper is a methodological innovation and does not involve retrievable data, measurement errors and other related data are already included in the paper. If the relevant code is needed, it can be obtained by contacting the authors via email.
